# Good knowledge about hypertension is linked to better control of hypertension; A multicentre cross sectional study in Karachi, Pakistan

**DOI:** 10.1186/1756-0500-5-579

**Published:** 2012-10-24

**Authors:** Aysha Almas, Saniya Siraj Godil, Saima Lalani, Zahra Aziz Samani, Aamir Hameed Khan

**Affiliations:** 1 Department of Medicine, Aga Khan University, Stadium road, Karachi, Pakistan; 2 Aga Khan Medical College, Karachi, Pakistan

**Keywords:** Hypertension, Knowledge, Uncontrolled hypertension

## Abstract

**Background:**

According to the National Health survey only 3% of the population has controlled hypertension. This study was designed to elucidate the knowledge about hypertension in hypertensive patients at three tertiary care centers in Karachi. Secondly we sought to compare the knowledge of those with uncontrolled hypertension and controlled hypertension.

**Methods:**

It was a cross-sectional study conducted at The Aga Khan University hospital (AKUH), Ziauddin Hospital (ZH) and Civil hospital, Karachi (CHK. All diagnosed Hypertensive patients (both inpatients and outpatients) coming to a tertiary care hospital in Pakistan aged > 18 years were included. Patients were categorized into 2 groups: controlled and uncontrolled hypertension based on their initial BP readings on presentation Uncontrolled Hypertension was defined as average BP ≥ 140/90 mm Hg in patients on treatment. Controlled Hypertension (HTN) was defined as average BP <140/90 mm Hg in patients on treatment. Standardized methods were used to record BP in the sitting position. Knowledge was recorded as a15 item question. Primary outcome was knowledge about hypertension.

**Results:**

A total of 650 participants were approached and consented 447 were found eligible. 284(63.5%) were from Aga Khan University, 101(22.6) from Dow University of health sciences and 62(13.9) were from Ziauddin University. Mean (SD) age of participants was 57.7(12) years, 50.1(224) were men. Controlled hypertension was present in 323(72.3) and uncontrolled hypertension was present in 124(27.4). The total mean (SD) Knowledge score was 20.97(4.93) out of a maximum score of 38. On comparison of questions related to knowledge between uncontrolled and controlled hypertension, there was statistically significant different in; meaning of hypertension (p <0.001), target SBP(p0.001), target DBP(p 0.001), importance of SBP versus DBP, improvement of health with lowering of blood pressure (p 0.002), high blood pressure being asymptomatic (p <0.001), changing lifestyle improves blood pressure(p 0.003),hypertension being a lifelong disease (<0.001), lifelong treatment with antihypertensives(<0.001) and high blood pressure being part of aging(<0.001). On comparison of knowledge as a composite score between uncontrolled and controlled hypertensive; Mean (SD) score was 21.85(4.74) v18.67 (4.70) (p value: < 0.001). On multivariate analysis; gender β (95% CI) 1.67(0.75, 2.59) p <0.001, uncontrolled blood pressure; -2.70(−3.76,-1.67) p <0.001, Sindhi ethnicity; -1.79(−3.25,-3.27) p 0.01 and pukhtoon ethnicity; -2.72(−4.13,-1.32) p <0.001 were significantly associated with knowledge score.

**Conclusion:**

Knowledge about hypertension in hypertensive patients is not adequate and is alarmingly poor in patients with uncontrolled hypertension. More emphasis needs to be made on target blood pressure and need for taking antihypertensives for life to patients by physicians.

## Background

Hypertension (HTN) is one of the most common medical disorders, associated with an increased incidence of all-cause and cardiovascular disease (CVD) mortality [1]. Fifty-four per cent of strokes and 47% of cardiac deaths are attributed to suboptimal blood pressure control [2]. Despite presence of a variety of antihypertensive medications hypertension remains uncontrolled [3]. Data on 22,282 patients, from national and regional blood pressure control and antihypertensive pharmacotherapy prescribed in cardiology practice quotes figure of overall 21.2% controlled hypertension. The control rates of hypertension in patients presenting to primary care range from 37% in Italy to 65% in South Africa and Canada [4-7]. According to a survey in 2010 control rates of hypertension are barely 6% in primary health care settings of Pakistan [8].

According to the National Health Survey of Pakistan (NHSP) the prevalence of HTN amongst adults >15 yrs was 17.9% and amongst them only 3% had their BP under control as opposed to a prevalence of 28.7% and control of around 31% being reported from US [9,10]. Hence there is sharp contrast in the control of hypertension in between the two countries. Several studies have been conducted to identify these factors and they have been broadly classified into patient-related and physician related factors. Physician related factors responsible for control of hypertension include practice patterns of the physicians, their knowledge base and perceptions about the care delivered by them [11].

The NHSP showed that 70% of all hypertensive in Pakistan are unaware if their condition [9]. Knowledge regarding control of hypertension including preventive mechanisms, factors affecting control of hypertension like diet, physical activity and insight into measures which can prevent complications of hypertension, have not been studied in depth in our population. There are studies done on medical students and physicians on their knowledge about hypertension but there is limited data from patients [12]. Some studies have been done on awareness in hypertensive patients in Pakistan, however these have mainly focused on risk factor identification for hypertension and not primarily on control of hypertension [13,14]. We therefore designed this study to elucidate the knowledge about hypertension in hypertensive patients at three tertiary care centers in Karachi. Secondly we sought to compare the knowledge of patients with uncontrolled hypertension and controlled hypertension.

## Methods

### Study design and population

It was a crossectional study conducted at three tertiary care hospitals in Karachi; Aga Khan Hospital, Ziauddin Hospital and Civil Hospital Karachi. The Aga Khan Hospital is a 577 bedded facility, which provides high quality of patient care in a broad range of secondary and tertiary services to over 50,000 hospitalised patients and to approximately 600,000 outpatients annually. It is located in the center of Karachi. The Ziauddin hospital is a 300 bedded hospital and is located in north of Karachi. The Civil hospital is a 1900 bedded public sector hospital located in center of Karachi. Population of different socioeconomic strata caters to these hospitals. The study was conducted in 2009–2010. All hypertensive patients (an average BP of ≥140/90 mm Hg or if the participant is taking antihypertensive medications) > 18 years of age presenting to the outpatient clinics were included [15]. Diagnosis of hypertension was confirmed by history, medical file or last prescription of any antihypertensive. Those who visited a doctor for the first time with hypertension were excluded. Patients were consecutively recruited from the outpatient setting of each hospital. Ethical approval was taken from the ethical review committee Aga Khan University (1193-Med/ERC-09). Ethical approval was also taken from Institutional Review board, Dow University of Health Sciences (IRB/DUHS/2010/40) and Ethics committee of Ziauddin University (letter 08-04-2010) Medical specialists conducting the clinic were informed about the conduct of the study. Patients fulfilling the inclusion criteria were approached and recruited by the trained research staff. Informed consent was taken from all patients before recruitment.

### Study variables and measurement

Primary outcome was knowledge score which was based on 15 item questions. Knowledge was also computed as a composite measure of sum of all the 15 item questions. These questions were developed by brainstorming with experts managing hypertension and who suggested that correct response of these questions can be counted towards having sound knowledge on hypertension (in patients). Questions regarding knowledge were asked and were marked on a scale of 1–5. (Additional file 1). Higher scores on a scale of 1–5 denoted more affirmative (positive) response and alluded to a better knowledge of hypertension There were some item questions which had multiple responses also. Each correct response is given a score of 1, and incorrect response is given score of 0, where there are multiple correct responses for one item question, score of 1 is give for each correct response. Sum of scores of each item question is given as total score=38. These questions were first piloted on a 20 patients (outside the study center) before using them in the study. Questions regarding adherence and physician related factors were also asked however they are not reported here. Each questionnaire took 15–20 minutes for completion.

Blood pressure was recorded twice at the time of recruitment in the right arm using a mercury sphygmomanometer with the individual in the sitting position. Average of these blood pressure readings was taken as the blood pressure reading for categorization into controlled and uncontrolled hypertension. Data was then recorded by the research staff on demographics, details on history of hypertension and antihypertensive and comorbids on a data collection form specifically designed for this purpose. (Additional file 1) Ethnicity was reported as ‘mother tongue’, which is specific for each of the five major ethnic subgroups of Pakistan: Urdu speaking, Punjabi, Sindhi, Pashtu, and Balochi. Formal education was categorized based on having no formal education, < 5 years, 6–10 years and > 10 years. A history of physician-diagnosed diabetes, stroke, ischemic heart disease (IHD), chronic kidney disease (CKD) as documented in the medical records was noted. Diabetes was defined as fasting plasma glucose ≥126 mg/dl at a prior visit [16]. Stroke was defined clinically as an acute neurologic dysfunction of vascular origin with sudden(within seconds) or at least rapid (within hours)occurrence of symptoms and signs corresponding to the involvement of focal areas in the brain [17]. Ischemic heart disease was diagnosed using WHO definition [18]. Smokers were defined as individuals who reported current smoking and having smoked at least 100 cigarettes or ‘birdees’ during their lifetime [19].

The Research staff was trained for a week to fill these data collection form. However the data collection form was not validated. Confidentiality was maintained and each data collection form was assigned a code which was kept under lock and key. To compensate for the time spent in filling the form, each patient was given an information brochure on hypertension. Data was entered by two separate data entry operators on epiinfo.

### Statistical analysis

Statistical package for social sciences version 17 was used for analysis. Mean and standard deviation was used for quantitative variable and frequency and percentage for qualitative variables. Knowledge is reported as frequency and percentage for individual correct response to each item question and composite total score; mean (SD). Data was also stratified on basis of control of hypertension. Uncontrolled hypertension was defined as average BP ≥140/90 mm Hg in no diabetic patients on treatment (for diabetic participants, hypertension will be considered to be uncontrolled if the average BP is ≥130/80 mm Hg) [15]. Controlled hypertension was defined as average BP <140/90 mm Hg in nondiabetic patients on treatment (for diabetic participants, hypertension will be considered to be uncontrolled if the average BP is <130/80 mm Hg). Student’s *t* test was used to compare quantitative variable and chi square test to compare categorical variable. Linear regressions were used to determine association of knowledge score with candidate variables. Multiple linear regressions were used to determine the association of all these variables in one model. Dummy variables were made for variables of ethnicity and level of education as they had more than two categories. All variable which were clinically important variables, or those that were associated with the outcomes at *P* < 0.2 in the univariate analysis were run in the final model. Final model was adjusted for age and duration of hypertension.

## Results

A total of 650 participants were approached; 350 in AKU, 150 in DUHS and 150 in ZMU.Of the total participants who were approached and who consented, 447 were found eligible%(n); 63.5(284) from Aga Khan University, 22.6(101) from Dow University of health sciences and 13.9(62) from Ziauddin University. Overall response rate was 71.52%; 81% in AKU, 80% in DUHS and 49.6% in ZMU.

Mean (SD) age of participants was 57.7(12) years, 224(50.1) were men and 223(49.8) were women. In ethnicity 108(24.3) were of Muhajir ethinicity. In education, 208(46.5) had > 10 years formal education. Comorbid conditions are shown in Table 1. Mean (SD) duration of hypertension was 9.9(7.42) year, Systolic blood pressure was 135.1(22.4), Diastolic blood pressure was 84.4(13.2). Controlled hypertension was present in 72.3%(323) and uncontrolled hypertension was present in 27.4%(124).

**Table 1:** Comparison of demographics and comorbids in patients with controlled and uncontrolled hypertension

**Characteristic**	**Overall%(n)**	**Controlled Hypertension%(n)**	**Uncontrolled Hypertension%(n)**	P value*
	**N**=**447**	**72.3%(323)**	**27.7(124)**	
**Demographics**				
Mean Age(SD)	57.7(12.0)	59.05(11.2)	54.33(13.4)	<0.001
Male gender	50.1(224)	38.3(171)	11.9(53)	0.03
Ethnicity				
*Sindhi*	15.9(71)	10.6(47)	5.4(24)	
*Punjabi*	11.9(53)	8.5(38)	3.4(15)	
*Balochi*	4(18)	2.2(10)	1.8(8)	
*Path an*	17.2(77)	11.2(50)	6.1(27)	
*Urdu speaking*	24.3(108)	19.6(87)	4.7(21)	
*Others*	26(118)	20.2(90)	6.2(28)	0.07
Level of education				
*No formal education*	32.2(144)	19.8(88)	12.6(56)	
<*5 yrs*	10.3(46)	7(31)	3.4(15)	
*6*–*10*	10.3(46)	6.8(30)	3.6(16)	<0.001
>*10 yrs*	46.5(208)	38.7(172)	8.1(36)	
**Comorbids**				
Diabetes	38(170)	27.1(121)	11(49)	0.38
Dyslipidimic	46(209)	38.2(167)	9.6(42)	0.002
Smoking	20.6(92)	17.3(77)	3.4(15)	0.004
Cerebro vascular accident	11.2(50)	7.5(33)	3.8(17)	0.16
Ischemic heart disease	32.4(145)	26.1(113)	7.4(32)	0.06

*p value between controlled and uncontrolled hypertension.

### Knowledge

Outcome of knowledge is reported as percentage(n) and mean(SD) scores have also been computed. Table 1. On computing knowledge scores for each item question, the total mean (SD) score was 20.97(4.93) out of a maximum score of 38.

### Comparison of knowledge in men and women

On comparison of individual item question in men and women%(n); 57.7(128) men and 35.5(78) women agreed that hypertension means high blood pressure (p value <0.001), 93.5(203) man and 92.5(205) women agreed that hypertension can be dangerous (p value 0.93), 79.4(177) men and 60.5(135) women said that systolic blood pressure (SBP) should be <140 mm hg (p value <0.001), 78.6(176) men and 58.3(130) women said that diastolic blood pressure (DBP) should be < 90 mm hg (p value <0.001), 26.7(58) men and 11.8(26) women said that both SBP and DBP are important to control (p value <0.001, 88.8(192) men and 91.2(197) agreed that lowering blood pressure improves health (p value 0.82), 45.8(99) men and 33(71) women agreed that high blood pressure can be asymptomatic (p value 0.006), 82.8(184)men and 84(186) agreed that changing lifestyle improves blood pressure (p value 0.5), 76(166) men and 73.3(162) women agreed that hypertension is a lifelong disease (p value 0.3), 77(174) men and 72.8(161) women agreed that antihypertensives have to be taken for life (p value 0.4), 57.3(126) men and 50.4(112) said that high blood pressure is a part of aging (p value 0.4) The mean (SD) knowledge score in men was 21.8(4.9) and in women was 20.07(4.8) (p value <0.001).

### Comparison of demographics and knowledge in patients with uncontrolled and controlled hypertension

On comparison of demographics of patients with controlled and uncontrolled hypertension, there was statistically significant difference in; Mean (SD) age (59.05 years (11.2) v 54.33 years (13.4)p <0.001), gender (38.3% males v 11.9% males p 0.03),> 10 years of formal education (38.7% v8.1% p <0.001). Comorbids and history of hospitalization is shown in Table 1.

On comparison of questions related to knowledge, there was statistically significant different in; meaning of hypertension (p <0.001), target SBP (p0.001), target DBP (p 0.001), importance of SBP versus DBP, improvement of health with lowering of blood pressure (p 0.002), high blood pressure being asymptomatic (p <0.001), changing lifestyle improves blood pressure (p 0.003), hypertension being a lifelong disease (<0.001), lifelong treatment with antihypertensives (<0.001) and high blood pressure being part of aging (<0.001) (Table 2).

**Table 2:** Comparison of knowledge about hypertension in patients with controlled and uncontrolled hypertension

**Characteristic**	**Overall%(n)**	**Controlled Hypertension%(n)**	**Uncontrolled Hypertension%(n)**	P value**
	**N**=**447**	**72.3(323)**	**27.7(124)**	
**Hypertension means**				
High blood pressure	46.1(206)	38.7 (171)	7.9 (35)	<0.001
**Hypertension is dangerous**				
Agree*	62.6 (280)	49 (214)	15.1 (66)	
Strongly agree*	28.6 (128)	20.6 (90)	8.7 (38)	-
**Systolic BP should be**				
<140 mm hg*	69.8 (312)	54.3 (242)	15.7 (70)	0.001
**Diastolic BP should be**				
<90*	68.5 (306)	53.2 (238)	15.2 (68)	0.001
**Which BP is more important**				
both*	18.8 (84)	16 (70)	3.2 (14)	0.003
**Lowering BP improves health**				
agree*	67.8 (303)	53.2 (230)	16.9 (73)	
strongly agree*	19.4 (87)	13.4 (58)	6.5 (28)	0.02
**High BP is asymptomatic**				
agree*	31.3 (140)	27.6 (119)	4.9 (21)	
strongly agree*	6.7 (30)	4.4 (19)	2.6 (11)	<0.001
**Changing lifestyle improvesBP**				
agree*	66.0 (295)	50.5 (223)	16.3 (72)	
strongly agree*	16.8 (75)	12.9 (57)	4.1 (18)	0.003
**HTN is a lifelong disease**				
agree*	51.9 (232)	43.1 (189)	9.8 (43)	
strongly agree*	21.5 (96)	16.4 (72)	5.5 (24)	<0.001
**Antihypertensives for life**				
agree*	51.9 (232)	42.7 (190)	9.4 (42)	
strongly agree*	23.0 (103)	17.1 (76)	6.1 (27)	<0.001
**High BP part of aging**				
agree*	44.3 (198)	38.7 (171)	6.1 (27)	
strongly agree*	8.9 (40)	6.1 (27)	2.9 (13)	<0.001
**Symptoms of hypertension**				
headache*	25.5 (371)	61.9 (273)	22.2 (98)	
SOB*	9.6 (140)	24.5 (108)	7.3 (32)	
Dizziness*	11.3 (164)	27.2 (120)	10 (44)	
Ghabrahat*^€^	18.4 (268)	46.3 (204)	14.5 (64)	
Sweating*	7.6 (111)	18.6 (82)	6.6 (29)	
Irritability*	9.7 (141)	26.3 (116)	5.7 (25)	
Chest pain*	7.1 (104)	15.9 (70)	7.7 (34)	
Blurred vision*	4.2 (61)	9.8 (43)	4.1 (18)	
**Lifestyle factors**				
Meds*	20.5 (367)	59.4 (265)	22.9 (102)	
Exercise*	14.9 (267)	44.4 (198)	15.5 (69)	
less stress*	11.3 (202)	32.1 (143)	13.2 (59)	
quit smoking*	5.9 (106)	17 (76)	6.7 (30)	
less salt intake*	20.4 (364)	59.6 (266)	22 (98)	
loose weight*	10.6 (190)	30.7 (137)	11.9 (53)	
change diet*	14.9 (266)	43.9 (196)	15.7 (70)	
reduce alcohol*	1.0 (18)	2.9 (13)	1.1 (5)	
**Organs effected by HTN**				
heart*	30.6 (280)	44.7 (200)	17.9 (80)	
eyes*	13.6 (124)	19.5 (87)	8.3 (37)	
brain*	27.5 (251)	40.5 (181)	15.7 (70)	
kidneys*	15.1 (138)	21 (94)	9.8 (44)	
**Food that increases BP**				
egg*	17.0 (245)	42.5 (177)	16.3 (68)	
mutton*	11.9 (171)	31 (129)	10.1 (42)	
fried food*	20.2 (291)	52.4 (218)	17.5 (73)	
beef*	16.4 (236)	42.1 (175)	14.7 (61)	
ghee*	23.0 (331)	58.7 (244)	20.9 (87)	
nuts*	11.4 (164)	30.3 (126)	9.1 (38)	

*Score of 1 for correct response, only correct responses have been reported here,**p value between controlled and uncontrolled hypertension^€^Ghabrahat (meaning feeling of doom,helplessness).

On comparison of knowledge as a composite score between uncontrolled and controlled hypertensive; Mean (SD) was 21.85(4.74) v18.67 (4.70) (p value :< 0.001) Knowledge score on most of individual item questions between the two group was significantly different (Table 3).

**Table 3:** Comparison of knowledge score in patients with controlled and uncontrolled hypertension

**Characteristic**		**Overall N**=**447**	**Controlled hypertension N**=**323**	**Uncontrolled hypertension N**=**124**	*P value
	**score**	**Mean(SD)**	**Mean(SD)**	**Mean(SD)**	
What does hypertension mean?	1	0.46(0.49)	0.52(0.49)	0.28(0.45)	<0.001
Is HTN dangerous for your health?	1	0.62(0.48)	0.66(0.47)	0.53(0.50)	0.01
What should be your systolic BP?	1	0.69(0.45)	0.74(0.43)	0.56(0.49)	<0.001
What should be your diastolic BP?	1	0.68(0.46)	0.73(0.44)	0.54(0.49)	<0.001
Which measure is more important?	1	0.18(0.39)	0.21(0.41)	0.11(0.31)	<0.001
Would lowering BP improve your health?	1	0.67(0.46)	0.71(0.45)	0.58(0.49)	0.12
Is high BP asymptomatic?	1	0.31(0.46)	0.36(0.48)	0.16(0.37)	<0.001
Can changing lifestyle lower your BP?	1	0.82(0.37)	0.86(0.34)	0.72(0.44)	<0.001
Do you think HTN is a lifelong disease?	1	0.73(0.44)	0.80(0.39)	0.54(0.50)	<0.001
Do you think you have to take antihypertensives lifelong?	1	0.74(0.43)	0.82(0.38)	0.55(0.49)	<0.001
Is high BP an unavoidable part of aging?	1	0.53(0.49)	0.61(0.48)	0.32(0.46)	<0.001
Symptom of HTN	9	3.25(2.01)	3.37(2.04)	2.96(1.91)	0.05
Factors associated with HTN	8	4.10(2.06)	4.11(2.04)	4.06(2.11)	0.80
Organs affected by HTN	4	3.38(1.82)	3.38(1.82)	3.38(1.81)	0.98
Food associated with hypertension	6	3.73(2.31)	3.89(2.32)	3.31(2.22)	0.01
Total score	38	20.97(4.93)	21.85(4.74)	18.67(4.70)	<0.001
Total percent score		55.18 %	57.50%	49.10%	

*p value between controlled and uncontrolled hypertension.

### Regression analysis

On univariate linear regression for outcome of knowledge score; β(95% CI) was 0.064(0.026,0.102) for age, 1.79(0.89,2.69) for gender,-1.93(−3.3,-0.5) for sindhi ethinicity, -1.66(−3.23,-0.84) for Punjabi ethinicity, -2.22(−4.64,0.18) for balochi ethnicity, -2.84(−4.23,-1.44) for pukhtoon ethinicity, -0.95(−2.21,0.31) for Muhajir ethinicity, -0.99(−2.5,0.54) for education up to <5 years,-1.14(−2.68,0.39) for < 10 years of education, 0.75(−0.48,2.03) for >10 years of education, 0.47(−0.51,1.46) for history of hospitalization for uncontrolled hypertension, -3.17(−4.15,-2.19) for having uncontrolled hypertension. However on multivariate analysis gender β(95% CI) 1.67(0.75,2.59) p <0.001,uncontrolled blood pressure; -2.70(−3.76,-1.67)p <0.001,sindhi ethnicity; -1.79(−3.25,-3.27) p 0.01 and pukhtoon ethnicity; -2.72(−4.13,-1.32) p <0.001 were significantly associated with knowledge score (Table 4).

**Table 4:** Univariate and Multivariate Analysis of demographic and disease related factors with Knowledge scores

**Variables**	**Univariate**		**Multi variety**	
	**Beta(95%CI)**	**P value**	**Beta(95%CI)**	**P value**
Age	0.06(0.02,0.10)	0.001	0.01(−0.03,0.05)	<0.001
Gender	1.79(0.89,2.69)	<0.001	1.70(0.77,2.64)	<0.001
Ethnicity				
*Sindhi*	−1.93(−3.36,-0.50)	0.008	−1.71	0.02
*Punjabi*	−1.66(−3.23,-0.08)	0.03	−1.32	0.10
*Balochi*	−2.22(−4.64,0.18)	0.07	−1.77	0.16
*Path an*	−2.84(−4.23,-1.44)	<0.001	−2.62	<0.001
*Urdu speaking*	−0.95(−2.21,0.31)	0.14	−0.97	0.13
Formal education				
<*5 yrs*	−0.99(−2.53,0.54)	0.20	-	-
*6*-*10*	−1.14(−2.68,0.39)	0.14	-	-
>*10 yrs*	0.75(−0.48,2.00)	0.23	-	-
Uncontrolled hypertension	−3.17(−4.15,-2.19)	<0.001	−2.71(−3.82,-1.67)	<0.001
Duration of disease	0.04(−0.02,0.10)	0.22	0.01(−0.05,0.08)	0.6
History of hospitalization	0.47(−0.51,1.46)		-	-

## Discussion

We are first to report a knowledge score in patients with hypertension belonging to three different tertiary care hospitals in Karachi, Pakistan. We found that overall knowledge scores in hypertensive patients was not up to the mark. Furthermore scores in patients with uncontrolled hypertension were significantly low. The lowest scores were obtained to questions related to importance of systolic blood pressure versus diastolic blood pressure, high blood pressure being asymptomatic and high blood pressure being an unavoidable part of aging. Factors associated with knowledge scores were male gender, having uncontrolled hypertension and ethnicity. Interestingly formal education did not show any relation with high knowledge scores in these hypertensive patients. This suggests that specific knowledge about disease is needed and just education alone may not suffice.

Sufficient knowledge about hypertension in patients has been associated with greater medication adherence and better blood pressure control [20]. Several knowledge based instruments have been tested for assessment of the same in hypertensive patients [21]. However only one has been reported with specific item questions, the Hypertension knowledge interview schedule which is validated for the Spanish population [22]. High blood pressure(HBP) Knowledge Test has been used as another validated instrument tested on Korean population. The item questions used in this test are comparable to what has been used in our study [21]. A significant proportion of Korean Americans answered incorrectly on items about HBP symptoms and diagnosis, HBP medications, harmful effects of HBP over time, and the relationship between BP and cold weather. Similar responses have been obtained from hypertensive patients in Pakistan (South Asia). The reason for this lack of knowledge among patients is definitely a cause of concern. Besides the fact that the physicians’ role has been critical, lack of proper health infrastructure, less resources and almost a non existing health education system through media are several other factors complicating the situation.

Gaps in knowledge have been reported with regard to risk factors associated with hypertension in 110 university students in a crossectional study conducted in Gulf region. Nationality, course of study, raised blood pressure, and history of diabetes showed significant association with good knowledge, however their net effect was not significant by the Adjusted Odds Ratio [12]. We report in this study the knowledge about hypertension in hypertensive patients belonging to three different hospital setups. In contrast we found significant association of knowledge with high blood pressure. The questions were focused not only on risk factors, but also on target blood pressure values, target organ damage, factors related to control of hypertension. This patient population with controlled hypertension comprised mainly of men, Urdu speaking ethnicity and those who were educated for more than 10 years. Previous studies have reported controlled hypertension to be associated with women [23,24]. Additionally the control rates in this patient population is much better than what has been earlier reported in the National health survey of Pakistan(NHSP) [9]. The main reason behind this is that this data is from tertiary care setting whereas the NHSP is from the population across Pakistan. Multiple reasons exist, which are responsible for these variable control rates of hypertension. These comprise of physicians `overestimate control rates and limited efforts to control BP at BP values close to normal, lack of awareness on the patient side and non compliance to antihypertensives [25,26].

Similar results have been reported from Karachi, Pakistan on hypertensive and normotensives [13,14]. The focus in them was also risk factors of hypertension and normotensives were also included in the study. We have shown that knowledge rather than awareness overall in this group of patients is limited, and even worse in patients who have uncontrolled hypertension. The factors related to this lower level of knowledge are multiple as shown in the current study. However, this patient population is not aware about the target blood pressure on treatment, that antihypertensives are to be taken for life and that control of both systolic and diastolic blood pressure is important.

Less than 75% of people prescribed drugs for hypertension continue using them after 6 months after initiation, and this is associated with an increased risk of hospitalization for cardiovascular problems [27]. Knowledge about target blood pressure among hypertensive patients is also inappropriate in the European high-risk population of coronary in patients [28]. A similar study conducted in aprimary care setting in United States concluded that setting target BPs may promote hypertension self-management in high-risk patient population [29]. This clearly indicates that apart from lack of appropriate health education, there seems to be a lack of transfer of knowledge by physicians to patients and requires exploration. Role of physicians has been critiqued in several studies in terms of lack of knowledge and practice of treating hypertension [30-32]. We found that factors associated with knowledge scores were male gender, having uncontrolled hypertension and ethnicity. The reasons of better scores in men could be limited opportunities available to women in a society with deep rooted gender bias against girls [33]. The knowledge scores show negative associations with all the ethnic groups in our study, however these were more pronounced for balochi and pukhtoon. The reason for this could be that both these are less developed compared to the rest.

The strength of this study is that it is multi centered study focusing on in depth analysis of knowledge regarding hypertension. It points out specific aspects on knowledge about hypertension, by taking care of which the hypertensives may have better blood pressure control. There are several limitations to this study. Firstly the results from this study cannot be generalized to the rural population in Pakistan. Hence similar studies need to me conducted in rural areas as well. Secondly the questionnaire used for knowledge assessment was not validated; however it was pretested before using. Thirdly blood pressure reading at a single occasion was used for classifying patients into controlled and uncontrolled hypertension. Fourthly we did not account for white coat effect while making this classification. Although the research staff was trained in recording the responses of the item questions, the possibility of a small percentage of interviewer bias cannot be completely ruled out. Additionally we did not measure compliance to antihypertensive therapy in this patient population. However adherence related questions were asked and will be reported elsewhere.

## Conclusion

Knowledge about hypertension in hypertensive patients is not adequate and is alarmingly poor in patients with uncontrolled hypertension. More emphasis needs to be made on target blood pressure and need for taking antihypertensives for life to patients by physicians. Alternatively group education of such patients needs to be tested as an intervention to improve knowledge on these aspects in addition to the risk factors of hypertension.

## Competing interests

The authors’ declare that they have no competing interests.

## Authors’ contributions

AA was responsible for conceiving the idea, drafting the protocol and getting approvals, SSG, SL and ZAH were responsible for implementation data collection and entry of the study, AA and AAH were responsible in drafting, revising and proof reading of the study. All authors read and approved the final manuscript.

## Supplementary Material

Additional file 1 Appendix A. Click here for file
