# Supplementary material for: Good knowledge about hypertension is linked to better control of hypertension; A multicentre cross sectional study in Karachi, Pakistan
Source: BMC Res Notes. 2012 Oct 24;5:579. doi: 10.1186/1756-0500-5-579 (PMC3534478; doi:10.1186/1756-0500-5-579)
Supplement: Additional file 1 — Appendix A. [file 1756-0500-5-579-S1.docx]

**Appendix A**

| E. KNOWLEDGE |  | | | | | | | | | |
| --- | --- | --- | --- | --- | --- | --- | --- | --- | --- | --- |
| 1. What does hypertension mean? | 1. High BP | | 2. High stress | | | | | 3. Nervous condition | | |
|  | 4. Don’t know | | | | 5. Others:_______________ | | | | | |
| 1. Is HTN dangerous for your health? | 1. Strongly disagree | | | 2. Disagree | | | 3. Neither agree or disagree | | | |
|  | 4. Agree | | | 5. Strongly agree | | |  | | | |
| 1. What should be your systolic BP? | 1. >140 | 2. 140 | | | | 3. <140 | | | | 4. Don’t know |
| 1. What should be your diastolic BP? | 1. >90 | 2. 90 | | | | 3. <90 | | | | 4. Don’t know |
| 1. Which measure is more important? | 1. Systolic | 2. Diastolic | | | | 3. Both | | | | 4. Don’t know |
| 1. Would lowering BP improve your health? | 1. Strongly disagree | | | 2. Disagree | | | 3. Neither agree or disagree | | | |
|  | 4. Agree | | | 5. Strongly agree | | |  | | | |
| 1. Is high BP asymptomatic? | 1. Strongly disagree | | | 2. Disagree | | | 3. Neither agree or disagree | | | |
|  | 4. Agree | | | 5. Strongly agree | | |  | | | |
| 1. What are the symptoms of high BP? | 1. Headache | | | 2. Shortness of breath | | | | | 3. Dizziness | |
|  | 4. Ghabrahat | | | 5. Excessive sweating | | | | | 6. Irritability | |
|  | 7. Chest pain | | | 8. Blurred vision | | | | | 9. Nausea | |
| 1. Can changing lifestyle lower your BP? | 1. Strongly disagree | | | 2. Disagree | | | 3. Neither agree or disagree | | | |
|  | 4. Agree | | | 5. Strongly agree | | |  | | | |
| 1. Do you think HTN is a life long disease? | 1. Strongly disagree | | | 2. Disagree | | | 3. Neither agree or disagree | | | |
|  | 4. Agree | | | 5. Strongly agree | | |  | | | |
| 1. Do you think you have to take antihypertensives lifelong? | 1. Strongly disagree | | | 2. Disagree | | | 3. Neither agree or disagree | | | |
|  | 4. Agree | | | 5. Strongly agree | | |  | | | |
| 1. Is high BP an unavoidable part of aging? | 1. Strongly disagree | | | 2. Disagree | | | 3. Neither agree or disagree | | | |
|  | 4. Agree | | | 5. Strongly agree | | |  | | | |
| 1. What factors are important in controlling your BP?   (Can choose more than 1) | 1.Medications | 2. Exercise | | | | | | | 3. Less stress | |
|  | 4. Quit smoking | 5. Less salt intake | | | | | | | 6. Lose weight | |
|  | 7. Change diet | 8. Reduce alcohol | | | | | | | 9. Don’t know | |
| 1. Which organs can be affected by HTN?   (Can choose more than 1) | 1. Heart | 2. Eyes | | | | | | | 3. Brain | |
|  | 4. Kidneys | 5. None | | | | | | | 6. Don’t know | |
| 1. In your opinion, consumption of which foods will lead to increase in BP?   (Can choose more than 1) | 1. Egg | 2. Mutton | | | | | | | 3. Fried food | |
|  | 4. Beef | 5. Ghee | | | | | | | 6. Nuts | |
